# Supplementary material for: First Report of Phodopus sungorus Papillomavirus Type 1 Infection in Roborovski Hamsters (Phodopus roborovskii)
Source: Viruses. 2021 Apr 23;13(5):739. doi: 10.3390/v13050739 (PMC8145573; doi:10.3390/v13050739)
Supplement: Supplementary file 1 [file viruses-13-00739-s001.zip › viruses-1179242-supplementary.pdf]

SUPPLEMENTARY MATERIALS

Tabel S1: GenBank accession numbers of papillomavirus L1 genes and corresponding host cytochrome b nucleotide sequences, used in the phylogenetic analysis, sorted in the alphabetical order.

PV type	GenBank accession no. (of the L1 gene)
AplaPV1	MK620303
AsPV1	HQ625440
BgPV1	JX174437
BPV1	X02346
BPV2	M20219
BPV5	AF457465
BPV8	DQ098913
BPV13	JQ798171
BPV14	KP276343
BPV25	MG252779
CcaPV1	MT774140
CPV20	KT901797
EePV1	FJ379293
EsPV1	NC038518
EsPV2	KC858264
EsPV3	KC858265
FarcPV1	MK620302
HPV6	HG793870

Host	GenBank accession no. of the cytochrome b region
Anas platyrhynchos (mallard)	EU755253
Anas rubripes (American black duck)	AF059088
Apodemus sylvaticus (wood mouse)	AB033695
Bison bonasus (European bison)	KP866277
Bos grunniens (yak)	AB542193
Bos Taurus (cow)	D34635
Bubalus bubalis (water buffalo)	D88637
Canis lupus (dog)	EU352854
Capra ibex (Alpine ibex)	AF034735
Capreolus capreolus (roe deer)	KJ558325
Cervus elaphus (red deer)	AB001612
Eptesicus isabellinus (meridional serotine)	EU786824
Eptesicus serotinus (serotine bat)	MF038481
Equus asinus (common donkey)	FJ428527
Equus burchellii (plains zebra)	JF718888
Equus caballus (horse)	D82932
Erinaceus concolor (white-breasted hedgehog)	KF783121
Erinaceus europaeus (European hedgehog)	KF783119

HPV8	M12737
HPV16	LC456627
HPV18	LC509006
LsmiPV1	MK620304
LsmiPV2	MK620330
MaegPV1	MH616908
MaPV1	HG530538
MfPV3	EF558839
MfPV4	EF558841
MfPV5	EF558843
MfPV6	EF558840
MfPV7	EF558838
MfPV8	EF558842
MfPV9	EU490516
MfPV10	EU490515
MfPV11	GQ227670
MmiPV1	DQ269468
MmPV1	M60184
MmuPV1	GU808564
MnPV1	U01834
MscPV1	JQ814848
OaPV1	U83594

Felis catus (cat)	AB004238
Fratercula arctica (Atlantic puffin)	DQ385228
Giraffa camelopardalis (giraffe)	LT628417
Hippotragus niger (sable antilope)	AF022061
Homo sapiens (human)	U09500
Larus marinus (great black-backed gull)	AJ508140
Larus smithsonianus (American herring gull)	FM209896
Macaca fascicularis (long-tailed macaque)	AF295584
Macaca mulatta (rhesus macaque)	U38272
Mastomys natalensis (Natal multimammate mouse)	AF518342
Melanogrammus aeglefinus (haddock)	DQ020497
Mesocricetus auratus (Syrian hamster)	KY754035
Micromys minutus (harvest mouse)	AB033697
Miniopterus schreibersii (Schreibers's bat)	EF530348
Mus musculus (house mouse)	LC325158
Ovis aries (sheep)	D84205
Ovis gmelini (mouflons)	D84203
Pan paniscus (pygmy chimpanzee)	D38116
Pan troglodytes (common chimpanzee)	X93338
Papio hamadryas (hamadryas baboon)	AF295585
Peromyscus maniculatus (deer mouse)	MN124384
Phodopus roborovskii (Roborovski hamster)	GU797468

OaPV2	U83595
OaPV4	KX954121
PhPV1	JF304764
PmPV1	NC039039
PpPV1	X62844
*PpPV1[Cc]	AF020905
PsuPV1	HG939559
[a] PsuPV1 (APV 475)	MW602287
[b] PsuPV1 (APV 480)	MW602286
[c] PsuPV1 (APV 516)	MW602285
RalPV1	KT626573
RfPV1	NC038527
RnPV1	GQ180114
SsPV1	EF395818

Phodopus sungorus (Siberian hamster)	AF119266
Procyon lotor (raccoon)	GU175439
Rattus norvegicus (brown rat)	AB033713
Rhinolophus ferrumequinum (greater horseshoe bat)	AB085731
Rupicapra rupicapra (chamois)	AF034725
Rusa alfredi (spotted deer)	JN632698
Sus domesticus (pig)	AB015079
Tapirus bairdii (tapir)	JF718880

* PpPV1 isolated from a common chimpanzee.

[a,b,c] PsuPV1 isolates obtained in this study.

PV – papillomavirus; AplaPV – Anas platyrhynchos papillomavirus; AsPV – Apodemus sylvaticus papillomavirus; BgPV – Bos grunniens papillomavirus; BPV – Bos taurus papillomavirus; CcaPV – Capreolus capreolus papillomavirus; CPV – Canis familiaris papillomavirus; EePV – Erinaceus europaeus papillomavirus; EsPV – Eptesicus serotinus papillomavirus; FarcPV – Fratercula arctica papillomavirus; HPV – human papillomavirus; LsmiPV – Larus smithsonianus papillomavirus; MaegPV – Melanogrammus aeglefinus associated papillomavirus; MaPV – Mesocricetus auratus papillomavirus; MfPV – Macaca fascicularis papillomavirus; MmiPV – Micromys minutus papillomavirus; MmPV – Macaca mulata papillomavirus; MmuPV – Mus musculus papillomavirus; MnPV – Mastomys natalensis papillomavirus; MscPV – Miniopterus schreibersii papillomavirus; OaPV – Ovis aries papillomavirus; PhPV – Papio hamadryas papillomavirus; PmPV – Peromyscus papillomavirus; PpPV – Pan paniscus papillomavirus; PsuPV – Phodopus sungorus papillomavirus; RalPV – Rusa alfredi papillomavirus; RfPV –Rhinolophus ferrumequinum papillomavirus; RnPV – Rattus norvegicus papillomavirus; SsPV – Sus scrofa papillomavirus.
